# Method comparison of beta‐hydroxybutyrate point‐of‐care testing to serum in healthy children

**DOI:** 10.1002/jmd2.12245

**Published:** 2021-08-22

**Authors:** Komalben Parmar, Maua Mosha, David A. Weinstein, Rebecca Riba‐Wolman

**Affiliations:** ^1^ Department of Pediatric Endocrinology Connecticut Children Medical Center Hartford Connecticut USA; ^2^ Department of Pediatrics University of Connecticut School of Medicine Farmington Connecticut USA; ^3^ Department of Research Connecticut Children Medical Center Hartford Connecticut USA

**Keywords:** beta‐hydroxybutyrate, ketone comparison, ketotic hypoglycemia, point of care, precision Xtra ketone meter

## Abstract

Ketone production is a physiological phenomenon that occurs to avoid irreversible neurological damage from hypoglycemia, thereby serving as a marker of metabolic stress. The primary ketone body, beta‐hydroxybutyrate (BHB), guides the diagnostic evaluation and management of many hypoglycemic disorders. Serum and point‐of‐care (POC) BHB values were not been compared in children without diabetes or metabolic disorders. We aim at comparing the serum and point‐of‐care BHB values in healthy children after an overnight fast. Eligible participants were ≤18 years of age prospectively recruited from elective procedures through our surgery centers. Exclusion criteria included a history of diabetes, hypopituitarism, adrenal, metabolic or inflammatory disorders, dietary restrictions, trauma, or use of medications that might affect blood glucose. The main outcome measure was comparing serum and POC BHB levels after a period of fasting. Data from 94 participants (mean age 8.29 ± 5.68 years, 54.3% male, 45.7% female, BMI mean 19.28 ± 5.25 kg/m^2^) were analyzed. There was a strong correlation between serum BHB (mean 0.25 ± 0.23 mmol/L) and POC BHB (mean 0.18 ± 0.20 mmol/L) (*r*_s_ = 0.803, *p* < 0.0001). The majority (96.81%) of values for serum BHB compared with POC BHB fell within 0.1 ± 0.1 mmol/L. The average of difference between serum and POC BHB (the bias) was 0.064 mmol/L (95% CI 0.047–0.081), and percentage error was 3.19%. Point‐of‐care BHB is accurate and comparable to serum BHB levels in our cohort of children after an overnight fast.

**Synopsis:**

Point‐of‐care BHB agrees with serum values in healthy children.

## 1 BACKGROUND

Ketone production is a physiological phenomenon that occurs as a result of fatty acid oxidation. Ketones build up from the accumulation of acetyl CoA, and they are a critical physiologic energy source. They can serve as an alternative energy source to the brain in the setting of hypoglycemia, thereby preventing neurologic damage and seizures.^1^ The measurement of ketones is standard of care in the management of patients with diabetes, but use in other disorders of carbohydrate metabolism has been more limited. There are several critical differences between urine and blood ketones. Ketone bodies consist of acetone, acetoacetate, and beta‐hydroxybutyrate (BHB). Acetoacetate is the primary ketone excreted in urine, whereas BHB is the primary type of ketone body in blood in both physiologic and pathologic states.^2^ During pathologic states, acidosis results in shunting of acetoacetate to BHB, and urine ketone measurements may underrepresent the state of ketosis due to the dilution effect and reflection of ketosis from the past. The detection and measurement of ketone levels is helpful during the evaluation and management of hypoglycemia.^3^, ^4^

Different modes of testing for BHB are available including laboratory‐based serum evaluation, which is considered the gold standard, and commercially available point‐of‐care testing (POCT). Comparison studies of these modalities in animals^5^, ^6^ and in humans with diabetes^7^ are available, which show that they are comparable. However, comparison studies in pediatrics patients without diabetes do not exist. The increasing accuracy and availability of POCT BHB monitoring has the possibility to become a central part of the diagnostic process in evaluating children with potential hypoglycemic disorders, as well as the ability to titrate their therapy to achieve control within the normal range. Therefore, the method comparison of POCT BHB against modern serum measurements is essential to determine accuracy and reliability. Our aim is to compare the commercially available POCT BHB and serum BHB values in children without known endocrine or metabolic disorder after an overnight fast.

## 2 MATERIALS AND METHODS

### 2.1 Patient selection

The Institutional Review Board (IRB) of Connecticut Children's Medical Center approved this study. One hundred participants were recruited who were scheduled to undergo elective surgeries at Connecticut Children's main campus and ambulatory surgical centers from July 1, 2020 until November 31, 2020, in cooperation with the surgical departments of otolaryngology, orthopedics, urology, and general surgery. Inclusion and exclusion criteria were defined. Children of ≤18 years of age undergoing elective surgeries per standard operating protocol (tonsillectomy and/or adenoidectomy, circumcision [new or revision], meatotomy, hernia repair, hydrocele repair, varicocele repair, orchiopexy, hypospadias repair, nonacute orthopedic surgeries) who required peripheral intravenous (IV) line placement were eligible. Ability and willingness to provide written informed consent from a legal guardian were necessary for study participation. Assent of a participant was sought, when appropriate, based on age and cognitive status. Children with serious medical and/or surgical conditions with comorbidities, diabetes, metabolic or mitochondrial disorders, adrenal gland disorders, suspected or diagnosed hypopituitarism, emergency surgeries, a history of enteral or parenteral steroid use in the previous 30 days, and dietary restrictions, or those on specialized diets (e.g., ketogenic diet) and those who drank any carbohydrate‐containing liquid on the day of surgery were excluded from participation.

### 2.2 Study protocol

Our team obtained IRB approval to contact the legal guardian(s) of potential eligible participants over the phone prior to surgery, and they were provided with a brief description of the study to determine their willingness to participate. Interested participants and their legal guardian(s) met the investigator in the preoperative area where informed written consent was obtained. At the time of IV placement (before intravenous fluid administration), 3–4 ml of whole blood was collected. Point‐of‐care testing (POCT) BHB was performed immediately (0.5 ml of unclotted whole blood at room temperature). The blood was collected in a gold‐top serum separator tube. To allow for clot formation, the samples were kept upright for 30 min at room temperature prior to centrifugation for 15 min at 3000 RPM (equivalent to 1008 RCF). Once centrifuged, serum was transported at room temperature within 12 hr and processed within 24 hr of the blood collection at the University of Connecticut Health Center, Department of Laboratory and Pathology Medicine. In addition to the sample of whole blood, participant demographic information was collected, including age, procedure performed, current medical diagnosis, date and time of last food/drink ingested, medication history, anthropometrics (height (cm), weight (kg), BMI (mg/m^2^) and), and BMI standardized for age (*z*‐score)) and date and time of blood draw.

POCT BHB measurements were performed using a precision Xtra meter (Abbott Pharmaceuticals), which displays BHB concentrations to a tenth of a unit in mmol/L, with a reported assay range of 0 to more than 8 mmol/L. It uses an enzymatic measurement with hydroxybutyrate dehydrogenase (HBDH). The precision Xtra meter was calibrated with Abbott MediSense Glucose and ketone control solutions as per the manufacturer's instructions. Serum BHB was measured by an Abbott Architect Chemistry System using the same method of enzymatic measurement with BHB dehydrogenase at a CLIA‐certified laboratory. Serum glucose was measured by spectrophotometry. The coefficient of variation (COV) obtained for the laboratory method for serum BHB is 0.99 and for the precision Xtra ketone meter is 0.96.^8^

### 2.3 Statistical analysis

Baseline characteristics and primary outcomes were analyzed using mean (±SD) and/or median (IQR). Spearman rank correlation was used for correlation analysis. A *p*‐value of less than 0.05 was considered statistically significant. The one‐sample Kolmogorov–Smirnov test and Bland–Altman plot analysis were used to assess whether the difference in the two methods of measurement was large enough to be systematic and whether there was agreement through the different levels of measurement. Maximum accepted difference was defined ahead of time as 0.2 between POCT and serum BHB (based on varying factors of clinical, biological, and other considerations). In order to ensure sufficient power of 90% for testing the reliability of the two methods of interest used to measure all the participants, two sets of sample sizes were calculated. For 90% power, an estimated mean difference of 0.1 ± 0.039 mmol/L and a max allowed difference of 0.2 mmol/L would require a minimum of 92 paired samples for Bland–Altman plot analysis.

## 3 RESULTS

### 3.1 Participant characteristics

One hundred participants were recruited for our study, of whom six were excluded from analysis (four did not meet the criteria, and two paired data of serum and POCT BHB were not available). Samples of 94 participants were analyzed (see Table 1 for baseline characteristics of the participants). The age of the participants ranged from 6 months to 18.7 years with a median (IQR) of 5.9 (3.6–13.6) years. Fifty percent (47/94) of patients received medication (*n* = 26 acetaminophen, *n* = 8 supplements, *n* = 3 epilepsy medication, *n* = 3 allergy medication, *n* = 2 psychiatric medications, *n* = 2 antacids, *n* = 1 for antibiotic, stimulant, and anti‐inflammatory nonsteroid medication). Serum BHB ranged from 0 to 1.2 mmol/L, whereas POCT BHB ranged from 0 to 1.1 mmol/L. Serum glucose ranged from 70 to 121 mg/dl. The rest of primary outcomes are listed in Table 2.

**TABLE 1:** Baseline characteristics of study participants

	*N* = 94 (%)	Mean ± SD
Age (years)		8.3 ± 5.7
BMI (kg/m^2^)		19.3 ± 5.2
Standardized BMI (*z*‐score)		0.50 ± 1.1
Duration of fast (hours)		12.5 ± 2.4
Sex		
Male	51 (54.3)	
Female	43 (45.7)	
Type of procedure		
Otolaryngology	43 (46)	
Orthopedic	20 (21)	
Urology	16 (17)	
General surgery	15 (16)	
Race		
White	60 (64)	
African American	13 (13)	
Asian	4 (4)	
Native Hawaiian or PI	1 (1)	
Other	10 (10)	
Refused	5 (5)	
Unknown	1 (1)	
Ethnicity		
Non‐Hispanic	74 (79)	
Hispanic	16 (17)	
Refused	3 (3)	
Unknown	1 (1)	

**TABLE 2:** Primary outcome of study participants

Variable (unit)	Mean ± SD	Median (IQR)
Serum beta‐hydroxybutyrate (BHB) (mmol/L)	0.25 ± 0.23	0.15 (0.10–0.40)
POCT BHB (mmol/L)	0.18 ± 0.20	0.10 (0.10–0.20)
Serum glucose (mg/dl)	90 ± 9.5	89 (83–95)

### 3.2 Comparison of POCT BHB and serum BHB

**FIGURE 1:**
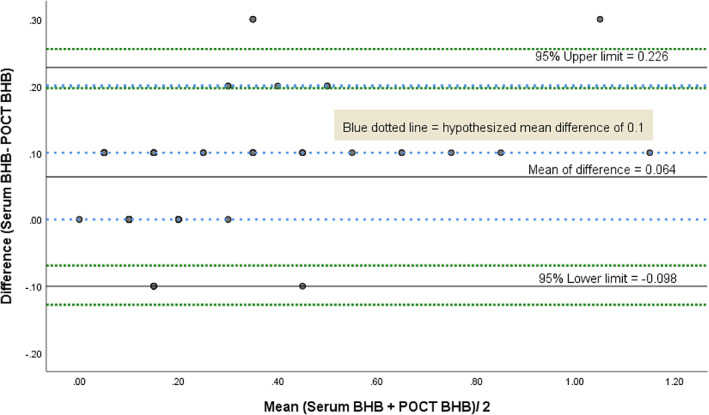
Bland–Altman plot analysis for method comparison between serum BHB and point‐of‐care testing (POCT) BHB

Correlation between serum BHB (mean 0.25 ± 0.23 mmol/L) and POCT BHB (mean 0.18 ± 0.20 mmol/L) was strong (*r*_s_ = .803, *p* < 0.0001), indicating a strong positive relationship (Figure S1) using the Spearman's rank test, but this alone does not necessarily depict agreement between two methods. The one‐sample Kolmogorov–Smirnov test indicated that the difference between serum BHB and POCT BHB measurement was not normally distributed (*p* < 0.0001). When we visualize this difference using Bland–Altman plot analysis (Figure 1), 96.81% of the values (91/94) fell within the hypothesized (blue dotted lines) margins of the mean difference of 0.1 ± 0.1 mmol/L. The average of difference between serum and POCT BHB (the bias) was 0.064 mmol/L (95% CI 0.047–0.081), which suggests on average serum BHB measured 0.064 (±0.083) points higher than POCT BHB, which was nonzero. In addition, the majority of the values were on the upper side of the zero line (positive difference between serum and POCT BHB). We have incorporated limits of maximum acceptable difference (0.2 mmol/L), based on whether the values fell within 95% CI of the upper and lower limits (represented by green dotted lines). In our study, the percentage error was 3.19%; 3/94 fall outside the upper limit of 0.25 difference (more precisely, three samples with a sampling difference of 0.3).

## 4 DISCUSSION

Ketone formation during periods of stress or counter‐regulation is a normal phenomenon, but it is critical to be able to accurately measure ketone concentrations to distinguish between physiologic and pathologic conditions. Measurement of urine ketones is limited by being qualitative, and pathologic ketosis is predominantly due to elevated BHB, which can be missed on present‐day urine testing. Prior studies on the portable ketone meter have focused on pathologic concentrations in the diabetes population with a focus on the higher concentrations. In the present study, ketone concentrations were measured in a cohort of healthy children without diabetes or other known endocrine or metabolic disorders after an overnight fast. The portable meter was found to have outstanding accuracy and precision in the concentrations more likely seen in nondiabetes disorders of carbohydrate metabolism.

In the context of illness, young children may develop both hypoglycemia and elevated ketone levels (ketotic hypoglycemia). Ketotic hypoglycemia can also be a defining feature of hypoglycemia seen in hormonal deficiencies (cortisol, growth hormone) and some metabolic disorders, including glycogen storage disease (GSD). In the absence of known metabolic or endocrine abnormalities, young children are given a diagnosis of idiopathic ketotic hypoglycemia. Ketones without associated hypoglycemia (euglycemic ketonemia) have been demonstrated in metabolic disorders, including hepatic GSDs, and may identify underlying metabolic stress prior to the onset of hypoglycemia.^9^, ^10^ Quantitative ketone measurements are useful in some metabolic disorders like GSD IV and IX, where monitoring may guide the management.^11^ It is also useful in differentiating disorders of glucose metabolism and other inborn errors of metabolism, such as disorders of fatty acid oxidation defects, ketone utilization disorders, and other metabolic disorders.^12^, ^13^

Diagnostic fasting studies that are often performed over 8–72 hr in order to assess both the ability to maintain euglycemia, as well as the metabolic responses to hypoglycemia, are the gold standard for the evaluation of hypoglycemic disorders. Fasting studies are labor‐intensive, expensive, and complex, and require that patients be hospitalized. Yet, fasting studies are frequently essential to identifying the diagnosis, which they do, in part, by differentiating between ketotic and hypo‐ketotic hypoglycemia. In children undergoing evaluation for hypoglycemic disorders, having a home monitoring tool for ketones may help to guide and further stratify clinical decision‐making (e.g., need for in‐patient fasting studies and genetic testing). The method comparison of two currently available modalities for BHB testing in a pediatric nondiabetic population is essential to develop more effective strategies for screening, diagnosis, and safe management of pathological causes of hypoglycemic disorder in children.

Reference values for an overnight fast in healthy children do not exist. In this article, our aim is to validate the method of point‐of‐care meter to best support our goal of developing pediatric ranges (which may ultimately vary by age or other phenotypic criteria, such as BMI, duration of fast, or blood sugar). This initial step is essential to improve the understanding of ketotic hypoglycemia. This study was not designed to generate the reference values for an overnight fast in children, but a subsequent research work will provide the description of this population and identify the need for the development of pediatric reference ranges.

One potential limitation of our study is that it may not be fully representative of healthy children in the general population, given that participants were those undergoing elective procedures. To minimize selection bias, we did not recruit participants who were undergoing elective surgeries in which a higher proportion of patients might have underlying inflammation or illness. Furthermore, potential participants were excluded if they were taking medications that have known effects on glucose metabolism (e.g., steroids or insulin). We have only compared the precision Xtra ketone meter from Abbott Pharma. Currently, there are other FDA‐approved blood ketone POCT meters available in the United States (Nova max, Freestyle Precision Pro, Ketosens). Further comparison studies between POCT and serum BHB values utilizing other commercially available ketone meters should be undertaken. Our venous samples were evaluated for BHB concentration as whole blood via POCT and serum via laboratory analysis. A potential limitation is that BHB POCT can also be measured via a capillary sample, which is not reflected in our study. A prior small study demonstrated a significant correlation between BHB in a capillary sample and a serum sample in a different population.^14^ Previous diabetes ketone comparison studies have shown good agreement up to the level of 5 mmol/L. As expected, our study did not compare the higher ketones values, as healthy children in the absence of known endocrine or metabolic disease should not have ketones as high as in other disease states. Our study identifies a potential measurement bias since we demonstrate a higher serum BHB compared with POCT levels. We also identified three samples, which had a larger difference of 0.3 mmol/L compared to the predefined accepted difference of 0.2 mmol/L, for which we have not been able to identify any specific factor.

Future studies to define the normative ranges of fasting ketones in a pediatric population without diabetes are necessary to fully utilize and validate this tool. Ketotic hypoglycemia disorders can have euglycemic ketosis as a feature.^9^ Identifying the presence of euglycemic ketosis in the context of normal daily activities (e.g., after a normal overnight fasting state while asleep or between feeds) may bring to attention children at higher risk of underlying metabolic disorders, which could lead to more prompt diagnosis and treatment.

The increasing accuracy and availability of POCT BHB monitoring has the possibility to become a central part of the diagnostic process in evaluating children with potential hypoglycemic disorders. Our research expands the tools available to investigate potential underlying disorders in children with hypoglycemia. Our results support that POCT BHB values are comparable to serum BHB and have the potential to be used during the evaluation and management of hypoglycemic disorders. These findings support the need to further define the normative ranges of ketones in healthy children.

## CONFLICT OF INTEREST

Komalben Parmar, Maua Mosha, David A. Weinstein, and Rebecca Riba‐Wolman declare that they have no conflict of interest.

## FUNDING INFORMATION

This research was supported by Connecticut Children's Medical Center. This work is a part of a fellow's scholarly work product in a 3‐year fellowship program. The author(s) confirm(s) independence from the sponsors; the content of the article has not been influenced by the sponsors.

## INFORMED CONSENT

All procedures followed were in accordance with the ethical standards of the responsible committee on human experimentation (institutional and national) and with the Helsinki Declaration of 1975, as revised in 2000 (5). This research was approved by the Institutional Board Review of our institution. Informed consent of the legal guardian and/or participant was obtained prior to participation in the study.

## Supporting information


**Figure S1** Spearman's correlation between serum and point‐of‐caring testing (POCT) for beta‐hydroxybutyrate (BHB)

## Data Availability

Data can be made available on a reasonable request from authors.
