# Supplementary material for: Method comparison of beta‐hydroxybutyrate point‐of‐care testing to serum in healthy children
Source: JIMD Rep. 2021 Aug 22;62(1):85–90. doi: 10.1002/jmd2.12245 (PMC8574180; doi:10.1002/jmd2.12245)
Supplement: Supplementary file 1 — Figure S1 Spearman's correlation between serum and point‐of‐caring testing (POCT) for beta‐hydroxybutyrate (BHB) [file JMD2-62-85-s001.pdf]

Supplementary Figure 1: Spearman's Correlation between Serum and Point of Care Testing for beta-hydrobutyrate

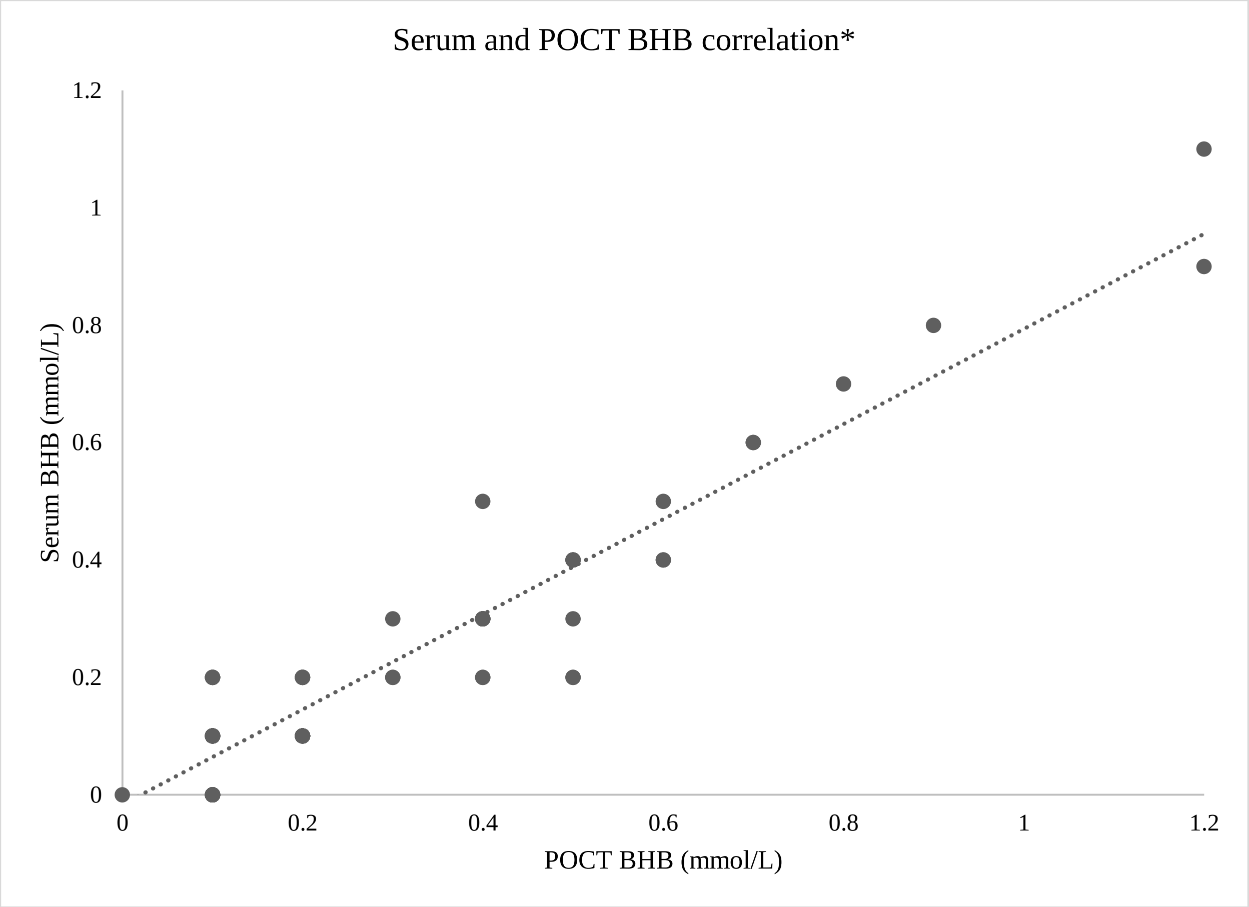

*Correlation coefficient (*r*) of 0.803, *p*<.0001, 2 tailed

Abbreviation: BHB; Beta-hydrobutyrate; POCT; Point of Care Testing
